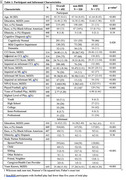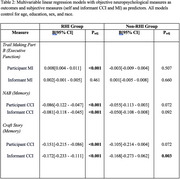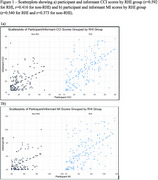# Characterizing Subjective Cognitive Complaints in Individuals Exposed to Repetitive Head Impacts

**DOI:** 10.1002/alz70857_106236

**Published:** 2025-12-26

**Authors:** Anna Aaronson, Caroline Altaras, Monica T. Ly, Jennifer Adler, Yorghos Tripodis, Steven Lenio, Chad William Farris, Christopher J. Nowinski, Maureen K. O'Connor, Brett Martin, Joseph N. Palmisano, Eric G. Steinberg, Katherine W. Turk, Rhoda Au, Lindsay A. Farrer, Gyungah R Jun, Lee E Goldstein, Wei Qiao Qiu, Andrew E. Budson, Thor D. Stein, Ann C. McKee, Jesse Mez, Michael L Alosco

**Affiliations:** ^1^ Boston University CTE and Alzheimer's Disease Research Center, Boston, MA, USA; ^2^ Boston University Chobanian & Avedisian School of Medicine, Boston, MA, USA; ^3^ Boston University Alzheimer's Disease Research Center, Boston, MA, USA; ^4^ Center of Excellence for Stress and Mental Health, VA San Diego Healthcare System, San Diego, CA, USA; ^5^ Boston University Chronic Traumatic Encephalopathy Center, Boston, MA, USA; ^6^ Department of Neurology, Boston University Chobanian & Avedisian School of Medicine, Boston, MA, USA; ^7^ Concussion Legacy Foundation, Boston, MA, USA; ^8^ Geriatric Research Education and Clinical Center, VA Bedford Healthcare System, Bedford, MA, USA; ^9^ Boston University Alzheimer's Disease Research Center & CTE Center, Boston, MA, USA; ^10^ VA Boston Healthcare System, Jamaica Plain, MA, USA; ^11^ The Framingham Heart Study, Boston University School of Medicine; Boston University School of Public Health, Boston, MA, USA; ^12^ Department of Epidemiology, Boston University School of Public Health, Boston, MA, USA; ^13^ Department of Anatomy & Neurobiology, Boston University Chobanian & Avedisian School of Medicine, Boston, MA, USA; ^14^ Biomedical Genetics, Department of Medicine, Boston University Medical School, Boston, MA, USA; ^15^ Alzheimer's Disease Research Center, Boston University Chobanian & Avedisian School of Medicine, Boston, MA, USA, Boston, MA, USA; ^16^ Department of Neurology and Ophthalmology, Boston University Chobanian & Avedisian School of Medicine, Boston, MA, USA; ^17^ Framingham Heart Study, Boston University Chobanian & Avedisian School of Medicine, Boston, MA, USA; ^18^ Department of Medicine (Biomedical Genetics), Boston University Chobanian & Avedisian School of Medicine, Boston, MA, USA; ^19^ Boston University Alzheimer's Disease Research Center, Boston, MA, USA; ^20^ Department of Psychiatry, Boston University Chobanian & Avedisian School of Medicine, Boston, MA, USA; ^21^ Alzheimer's Disease Research Center, Boston University Chobanian & Avedisian School of Medicine, Boston, MA, USA; ^22^ Department of Pharmacology & Experimental Therapeutics, Boston University Chobanian & Avedisian School of Medicine, Boston, MA, USA; ^23^ Department of Pathology and Laboratory Medicine, Boston University Chobanian & Avedisian School of Medicine, Boston, MA, USA; ^24^ VA Boston Healthcare System, Boston, MA, USA; ^25^ Boston University Chronic Traumatic Encephalopathy Center, Boston University Chobanian & Avedisian School of Medicine, Boston, MA, USA

## Abstract

**Background:**

Subjective cognitive complaints (SCCs) can be an early indicator of Alzheimer's disease and related dementias. SCCs have been shown to be common in people exposed repetitive head impacts (RHI), particularly male former professional American football players. This study characterized participant and informant‐reported SCCs in terms of rate, concordance with standardized neuropsychological measures, and potential associated factors among participants with diverse sources/severity of RHI exposure.

**Method:**

The sample included participants with (*n* = 172) and without (*n* = 320) RHI from the Boston University Alzheimer's Disease Research Center Clinical Core. RHI status is based on the 2021 NINDS TES Research Diagnostic Criteria. The Cognitive Change Index (CCI) and BRIEF‐A Meta‐Cognition Index (MI) measured self and informant‐reported SCCs. Participants completed neuropsychological assessments of memory (Craft Story 21 Recall, NAB List Learning Long Delay) and executive function (Trails B). Informants completed the Neuropsychiatric Inventory‐Questionnaire (NPI‐Q). ANCOVAs compared performance of RHI/non‐RHI groups on the SCC measures. Pearson correlation examined agreement between participant/informant responses. Multivariable linear regression models tested associations between SCCs and neuropsychological tests and examined correlates of SCCs. All models controlled for age, sex, race, and education. *p*‐values were false discovery rate adjusted.

**Result:**

Table 1 describes the sample. Compared to non‐RHI, the RHI group was younger, likelier to be male, and likelier to have MCI. The RHI group had significantly higher MI (B=8.084, *p* = 0.006), informant MI (B=9.014, *p* = 0.006), CCI (B=5.986, *p* <0.001), and informant CCI scores (B=7.062, *p* <0.001. Self/informant CCI and MI scores were more correlated in the RHI (*r* = 0.592, 0.540, respectively) vs non‐RHI group (*r* = 0.416, 0.373, respectively). Figure 1. Within the RHI group, there were associations between participant/informant SCCs and the objective measures (e.g., B=‐0.086, padj<0.001 for CCI and NAB) (Table 2). We observed fewer, weaker associations between SCCs and neuropsychological measures in the non‐RHI group. NPI‐Q was a consistent correlate of self/informant SCCs. Demographics (e.g., self/informant race) were also associated but to a lesser extent.

**Conclusion:**

In RHI settings, SCCs might be more frequent and reflect cognitive function. High SCC rates are likely multifactorial, with influence from neuropsychiatric factors. Future research should examine longitudinal change in self/informant SCCs and correlation with disease biomarkers.